# Proximal Tubular *Lats2* Ablation Exacerbates Ischemia/Reperfusion Injury (IRI)-Induced Renal Maladaptive Repair through the Upregulation of P53

**DOI:** 10.3390/ijms242015258

**Published:** 2023-10-17

**Authors:** Chi Zhang, Zhihuang Zheng, Kexin Xu, Guozhe Cheng, Huijuan Wu, Jun Liu

**Affiliations:** 1 Department of Nephrology, Shanghai General Hospital, Shanghai Jiaotong University School of Medicine, Shanghai 201600, China; lexiethegoat@sjtu.edu.cn (C.Z.); zhengz.sjtu.edu@gmail.com (Z.Z.); xkx2908@sjtu.edu.cn (K.X.); 15chenggz@sjtu.edu.cn (G.C.); 2 Laboratory of Nephropathy, Translational Medicine Center, Shanghai General Hospital, Shanghai Jiaotong University School of Medicine, Shanghai 201620, China; 3 Institute of Translational Medicine, Shanghai General Hospital, Shanghai Jiaotong University School of Medicine, Shanghai 201620, China; 4 Department of Pathology, School of Basic Medical Sciences, Fudan University, Shanghai 200030, China

**Keywords:** LATS2, p53, acute kidney injury, maladaptive repair, apoptosis

## Abstract

The Hippo pathway mediates renal maladaptive repair after acute kidney injury (AKI), which has been considered a driving force in the progression to chronic kidney disease (CKD). LATS2, a core kinase of the Hippo pathway, exerts non-Hippo-dependent functions in the regulation of the cell cycle and cell fate, providing new insights into AKI and further repair. However, its role remains unknown. Here, we utilized a proximal tubular *Lats2* conditional knockout mouse strain (*Lats2*-CKO) to evaluate the effect of LATS2 deficiency on ischemia/reperfusion-induced AKI-to-CKD transition. *Lats2*-CKO mice presented with more severe tubular maladaptive repair, inflammatory infiltration, interstitial fibrosis, and apoptosis following AKI. Importantly, we discovered that *Lats2* ablation caused the activation of p53, with increased levels of cellular apoptotic molecules (p21, Bax, and cleaved caspase-3), and decreased levels of anti-apoptotic molecules (Bcl-2 and Bcl-xL). Pifithirin-α (p53 inhibitor) effectively attenuated renal fibrosis, inflammation, and apoptosis in *Lats2*-CKO mice after AKI. Consistently, in vitro *Lats2* overexpression decreased p53, p21, Bax and cleaved caspase 3 expression after hypoxia/reoxygenation (H/R) treatment. Of note, the phosphorylation of MDM2, which promotes the ubiquitination degradation of p53, at site Ser186 was decreased in *Lats2*-CKO kidneys, but increased by *Lats2* overexpression in vitro. Therefore, LATS2 deficiency aggravated ischemia/reperfusion injury (IRI)-induced maladaptive repair via regulating the tubular MDM2-p53 axis in AKI-to-CKD transition.

## 1. Introduction

Acute kidney injury (AKI), characterized by a rapid decrease in glomerular filtration rate, is a common and destructive pathological condition [1]. AKI has a substantial impact morbidity and mortality [2], and incomplete recovery, also called maladaptive repair, can lead to long-term functional deficits and chronic kidney disease (CKD) [3,4]. The transition from adaptive to maladaptive kidney repair can be attributed to cytokine secretion, inflammation, myofibroblast generation, extracellular matrix (ECM) production, pathological apoptosis, and G2/M cell cycle arrest [5,6]. Therefore, defining the cellular and molecular pathways during the repair process after AKI can help identify novel therapeutic targets to prevent the transition to CKD.

The mammalian Hippo pathway is an evolutionarily conserved serine–threonine kinase cascade that plays an important role in cell growth and fate determination, organ size and regeneration [7,8]. The Hippo pathway is composed of core kinases, including MST1/2 and LATS1/2, and nuclear transcription co-activators YAP/TAZ [8]. Chen J et al. and our previous research have uncovered that Hippo-YAP plays a regulatory role in the pathogenesis and repair of AKI and CKD progression [9,10,11]. Regarding its classical function, LATS2 primarily participates in cellular activities by regulating the downstream effectors YAP/TAZ. However, recent studies have indicated that LATS2 can independently regulate the cell cycle and pathological apoptosis without the mediation of YAP/TAZ [12,13]. Traditionally, upon Hippo pathway activation, LATS1/2 are phosphorylated by MST1/2 [14]. The activated LATS1/2, together with MOB1, subsequently phosphorylate and deactivate YAP/TAZ through cytoplasmic sequestration and proteasomal degradation, ultimately governing cell growth and fate determination [15,16]. In cardiac research, it was found that LATS2 is activated during pressure overload, thereby contributing to heart failure by stimulating p53-dependent mechanisms of cell death [17]. However, the role of LATS2 independent of YAP/TAZ and its detailed mechanism in AKI-induced renal maladaptive repair remain unclear.

Furth et al. showed that downregulation of LATS can convert p53 from a tumor suppressor into a tumor facilitator in breast cancer [18]. Importantly, p53, a tumor suppressor, plays a critical role in AKI and subsequent kidney repair by orchestrating specific cellular responses, including cell cycle arrest, cellular senescence and apoptosis [19]. Recent studies have shown that p53 in renal proximal tubular cells promotes AKI by promoting apoptosis and inflammation [20,21]. Emerging evidence further suggests that p53 mediates the progression of renal fibrosis and chronic inflammation in maladaptive kidney repair after AKI [22,23,24]. Therefore, it is worth investigating whether LATS2 is involved in renal maladaptive repair of post-AKI by interacting with p53.

In this study, we utilized a conditional knockout mouse strain in which *Lats2* was specifically ablated in proximal tubular cells to explore the role of LATS2 in a model of maladaptive kidney repair after severe kidney injury (unilateral ischemia/reperfusion injury [U-IRI]). Strikingly, we found that LATS2 deficiency aggravated renal fibrogenesis, inflammatory infiltration, and apoptosis after severe ischemic AKI. Furthermore, in combination with *Lats2* modulation in vitro, the MDM2-p53 axis was found to mediate the effect of *Lats2* in post-IRI kidney maladaptive repair.

## 2. Results

### 2.1. RNA-seq Analysis Identified Downregulation of Lats2 in Post-IRI Kidneys

**Figure 1:**
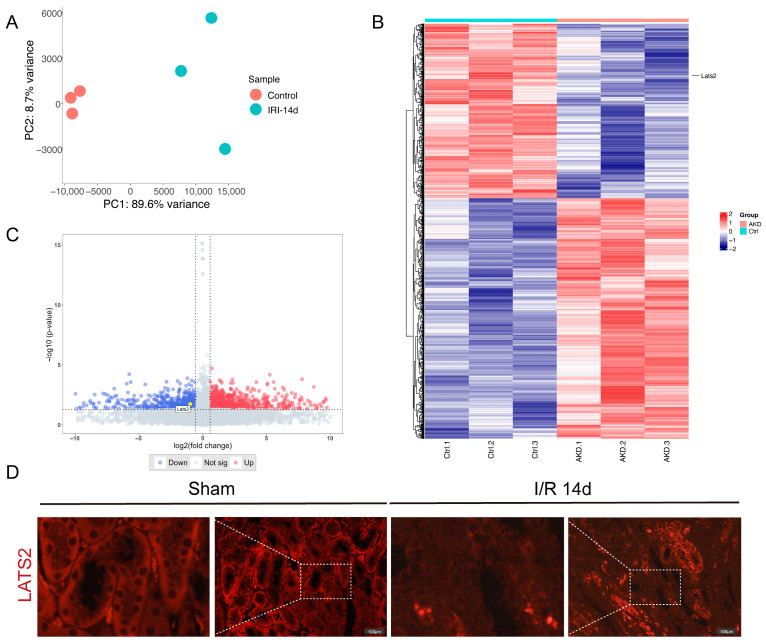
LATS2 expression changes after IRI through RNA-seq. (**A**) Principal component analysis (PCA) of the gene expression profiles. (**B**) Heatmap of differentially expressed genes (DEGs) from kidney tissues after IRI and controls. (**C**) A volcano plot of DEGs from kidney tissues after IRI and controls. (**D**) Representative immunofluorescence and enlarged images of LATS2 staining in sham and IRI-injured kidneys. Scale bars, 100 μm.

To evaluate *Lats2* expression in post-IRI kidneys, we conducted total mRNA sequencing of whole-kidney samples of three biological replicates with or without U-IRI. Principal component analysis (PCA) showed that the gene transcriptional profiles of post-IRI kidneys and controls were distinct from each other (Figure 1A). We then analyzed RNA-seq data at the gene level by quantifying fragments per kilobase of transcript per million mapped reads (FPKM) values from 51,811 genes. We identified 1592 differentially expressed genes (DEGs) with a threshold of |*logFC*| > 0.58 and an adjusted *p* value < 0.05. A heatmap and volcano plot were generated to present the expression pattern of DEGs, and *Lats2* mRNA was significantly downregulated after the IRI (Figure 1B,C). Next, we detected LATS2 expression in kidney tissues using immunofluorescence, the results indicating a substantial decrease in the expression of LATS2 in renal damaged proximal tubule epithelial cells (PTECs) following IRI (Figure 1D).

### 2.2. Loss of Lats2 Aggravates Kidney Parenchymal Injury in U-IRI Model

**Figure 2:**
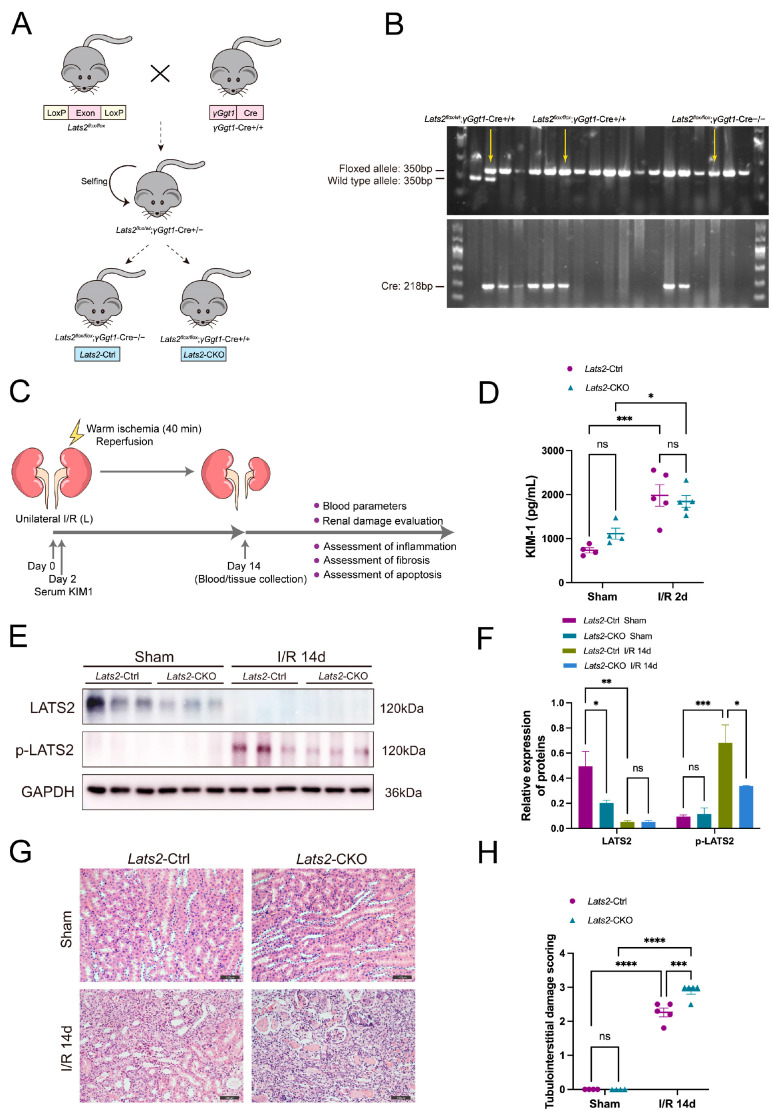
The effect of renal proximal tubule-specific *Lats2* ablation on kidney histopathology after I/R injury. (**A**) Schematic representation of *Lats2*-CKO mice established by crossing *Lats2*^flox/flox^ with *γGgt1*-Cre+/+ mice. (**B**) Identification of genotype *Lats2*^flox/flox^; *γGgt1*-Cre+/+ mice by PCR-based genotyping. (**C**) *Lats2*-Ctrl and *Lats2*-CKO mice were subjected to 40 min warm U-IRI. (**D**) Serum KIM-1 levels of mice were determined by ELISA in sham and U-IRI mice 2 days after U-IRI (sham *n* = 4, I/R 2d *n* = 5). (**E**) Western blotting analysis of LATS2, P-LATS2 (Ser872) in sham and IRI 14d mice and (**F**) quantified and normalized to GAPDH expression. (**G**) Representative HE staining of kidney in *Lats2*-Ctrl and *Lats2*-CKO mice subjected to U-IRI. Scale bars, 100 μm. (**H**) Semi-quantification of tubulointerstitial damage (sham *n* = 4, I/R 14d *n* = 5). Data expressed as means ± SEM. Two-way ANOVA followed by Tukey’s multiple comparisons post hoc test. * *p* < 0.05, ** *p* < 0.01 *** *p* < 0.001, **** *p* < 0.0001 defined as significant. ns, not statistically significant.

To test the role of LATS2 in renal repair after acute injury, we constructed a conditional knockout mouse model, in which *Lats2* was specifically deleted from PTECs. The breeding protocol is shown in Figure 2A. Briefly, *Lats2*^flox/flox^ mice were crossed with *γGgt1*-Cre+/+ mice. After the first round of breeding, heterozygous progenies (*Lats2*^flox/wt^; *γGgt1*-Cre+/−) were selected to further generate *Lats2*-CKO (*Lats2*^flox/flox^; *γGgt1*-Cre+/+, called *Lats2*-CKO hereafter) and *Lats2*-Ctrl (*Lats2*^flox/flox^; *γGgt1*-Cre−/−, called *Lats2*-Ctrl hereafter) littermate mice. The genotypes of the above mice were confirmed by PCR (Figure 2B). At the protein level, LATS2 expression level was significantly lower in *Lats2*-CKO kidney tissues than in *Lats2*-Ctrl tissues (Figure 2E). We then subjected 8-week-old male *Lats2*-CKO and *Lats2*-Ctrl mice to 40 min of U-IRI (Figure 2C). Serum KIM-1, a marker of proximal tubule injury, was markedly increased to similar levels in both groups at day 2 after U-IRI (Figure 2D), suggesting that the initial proximal tubule injury due to I/R was equivalent between *Lats2*-CKO and *Lats2*-Ctrl groups. Western blot showed decreased expression of LATS2, but increased phosphorylation of LATS2 at the site of Ser872 at 14 days post-AKI kidneys (Figure 2E,F). IRI-induced renal maladaptive repair mice showed abnormal tubules with progressive fibrosis and an accumulation of inflammatory cells, with *Lats2*-CKO mice displaying more severe tubulointerstitial damage than *Lats2*-Ctrl mice (Figure 2G,H). In addition, there were no significant differences in serum Cr or BUN levels between the groups at 2 or 14 days after U-IRI treatment and the sham group, or between *Lats2*-CKO mice and *Lats2*-Ctrl mice (Figure S1).

### 2.3. Proximal Tubule-Specific Knockout of Lats2 Exacerbates Renal Fibrosis after AKI

**Figure 3:**
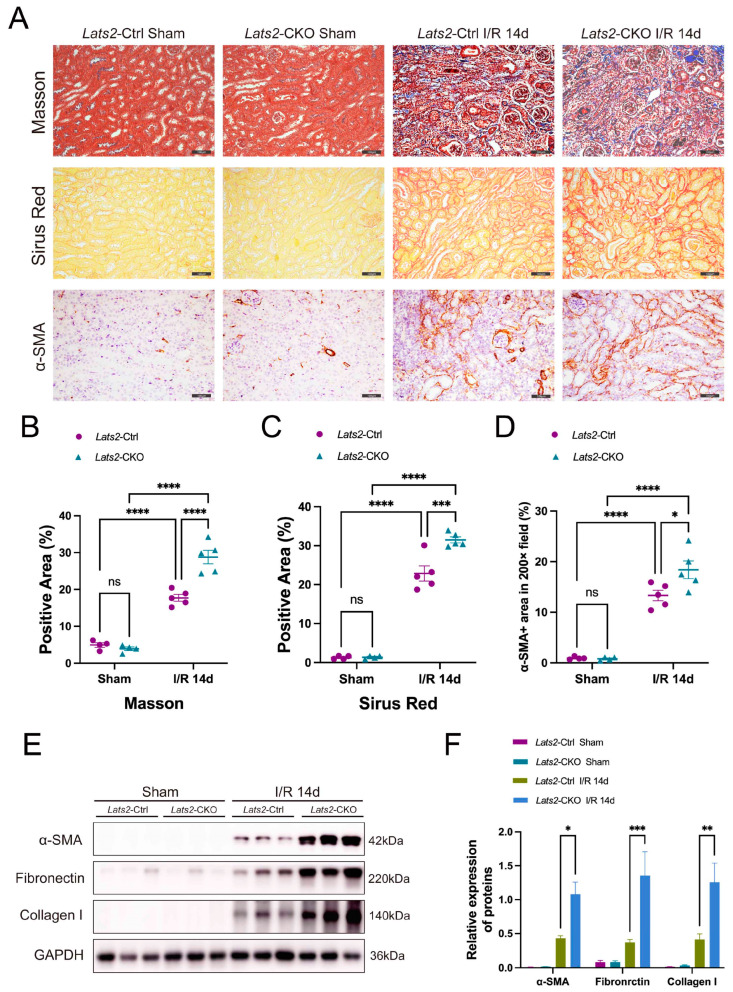
The effect of renal proximal tubule-specific *Lats2* ablation on renal fibrogenesis after I/R injury. (**A**) Representative images of sham and IRI-injured kidneys stained with Masson, Sirius red, and α-SMA. Scale bars, 100 μm. (**B**) Semi-quantification in renal Sirius red positive area proportion (sham *n* = 4, I/R 14d *n* = 5). (**C**) Semi-quantification in renal Masson positive area proportion (sham *n* = 4, I/R 14d *n* = 5). (**D**) Quantification of α-SMA+ staining (sham *n* = 4, I/R 14d *n* = 5). (**E**) Western blotting analysis of α-SMA, fibronectin and collagen I in sham and IRI 14d mice and (**F**) quantified and normalized to GAPDH expression. Data expressed as means ± SEM. Two-way ANOVA followed by Tukey’s multiple comparisons post hoc test. * *p* < 0.05, ** *p* < 0.01, *** *p* < 0.001, **** *p* < 0.0001 defined as significant. ns, not statistically significant.

Since maladaptive repair after IRI contributes to the gradual aggravation of renal fibrosis [25], we examined the influence of proximal tubule-specific *Lats2* ablation on renal fibrosis after AKI. A considerable increase in extracellular matrix (ECM) deposition was observed in IRI kidneys compared to sham kidneys, as demonstrated by Masson trichrome and Sirius red staining (Figure 3A–C). Proximal tubule-specific *Lats2* knockout significantly augmented this ECM deposition (Figure 3A–C). Similarly, compared to *Lats2*-Ctrl mice, *Lats2* knockout further augmented the increase in α-smooth muscle actin (α-SMA), fibronectin and collagen I in IRI kidneys (Figure 3A,D–F and Figure S2). The above data suggest that renal fibrosis is exacerbated in renal maladaptation in response to *Lats2* knockout.

### 2.4. Lats2 Deficiency Leads to More Renal Inflammatory Cell Infiltration and Apoptosis

**Figure 4:**
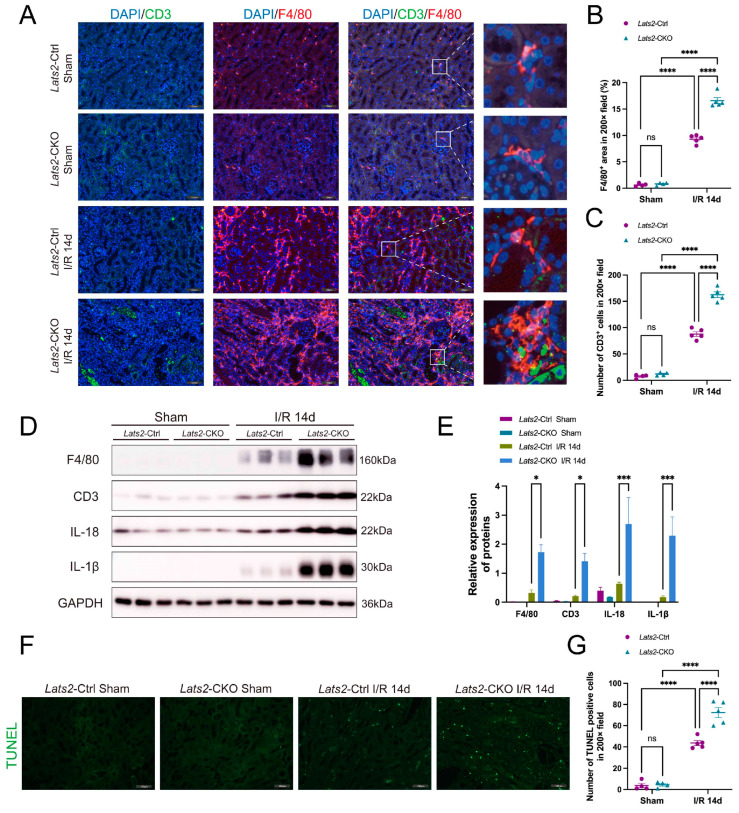
The effect of renal proximal tubule-specific *Lats2* ablation on inflammatory cell accumulation and apoptosis after I/R injury. (**A**) Representative dual fluorescence and enlarged images of CD3 and F4/80 staining in sham and IRI-injured kidneys. Scale bars, 100 μm. (**B**) Quantification in renal infiltration of CD3+ T cells (sham *n* = 4, I/R 14d *n* = 5). (**C**) Quantification in renal infiltration of F4/80+ macrophages (sham *n* = 4, I/R 14d *n* = 5). (**D**) Western blotting analysis of F4/80, CD3, IL-18 and IL-1β in sham and IRI 14d mice and (**E**) quantified and normalized to GAPDH expression. (**F**) Representative TUNEL staining of kidney in *Lats2*-Ctrl and *Lats2*-CKO mice subjected to I/R injury. Scale bars, 100 μm. (**G**) Quantification of TUNEL-positive cells in kidney (sham *n* = 4, I/R 14d = 5). Data expressed as means ± SEM. Two-way ANOVA followed by Tukey’s multiple comparisons post hoc test. * *p* < 0.05, *** *p* < 0.001, **** *p* < 0.0001 defined as significant. ns, not statistically significant.

AKI is typically accompanied by immune activation and inflammation [26]. Therefore, we examined whether the deficiency of *Lats2* on proximal tubules led to changes in renal inflammatory response. Macrophage marker F4/80 and T cell marker CD3 were detected using co-immunofluorescence. As shown in Figure 4A–C, the number of CD3-positive T cells and F4/80-positive macrophages in IRI kidneys was markedly increased compared to sham kidneys. Significantly, *Lats2*-CKO mice showed more severe I/R-induced macrophage and T-cell infiltration than *Lats2*-Ctrl mice. Furthermore, *Lats2*-CKO mice had higher protein expressions of CD3, F4/80, IL-18 and IL-1β compared to *Lats2*-Ctrl mice after IRI (Figure 4D,E).

Apoptosis plays a pathogenic role in ischemic AKI [27], and LATS2 has been implicated in the regulation of the cell cycle and apoptosis [28]. Therefore, we evaluated apoptosis in kidney tissues by TUNEL assay. As shown in Figure 4F,G, no apoptosis was observed in the kidney tissues from sham-operated mice. By the day of 14 after renal IRI treatment, significant apoptosis was found in kidney tissues. Notably, proximal tubule-specific *Lats2* knockout induced more pronounced apoptosis after IRI than the control group.

### 2.5. Proximal Tubule-Specific Lats2 Knockout Upregulates p53 Expression in Renal Maladaptive Repair

**Figure 5:**
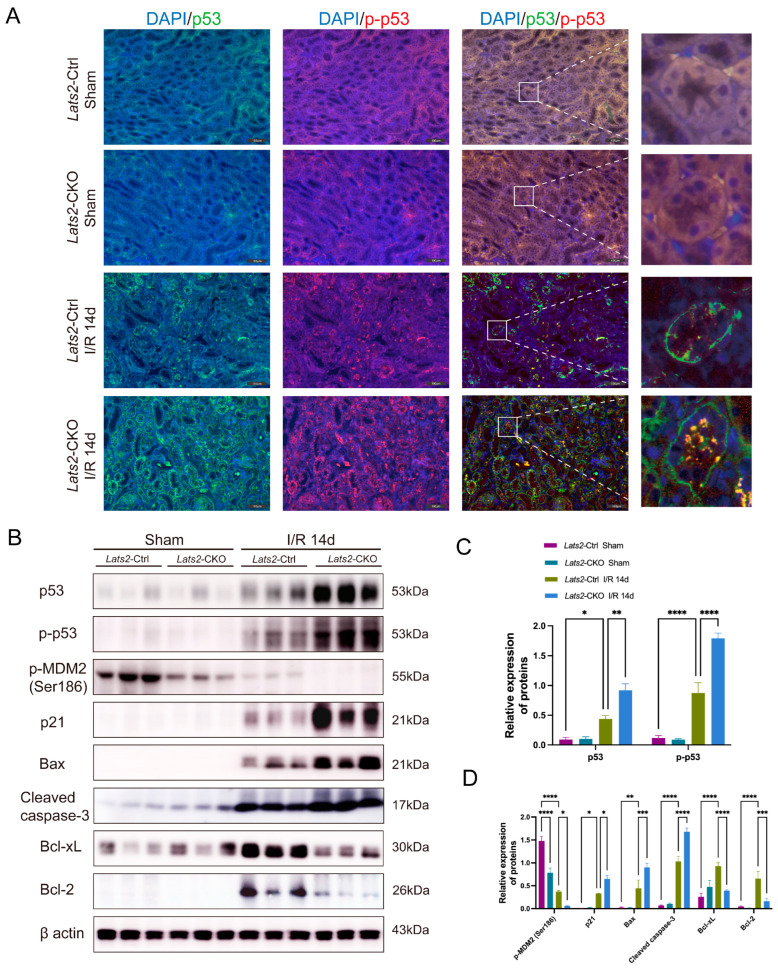
The effect of renal proximal tubule-specific *Lats2* ablation on p53 and its target gene expression after I/R injury. (**A**) Representative dual fluorescence and enlarged images of p53 and p-p53 (Ser 15) staining in sham and IRI-injured kidneys. Scale bars, 100 μm. (**B**) Western blotting analysis of p53, p-p53, p-MDM2 (Ser186), p21, Bax, cleaved caspase-3, Bcl-xL, and Bcl-2 in sham and IRI 14d mice and (**C**,**D**) quantified and normalized to β-actin expression. Data expressed as means ± SEM. Two-way ANOVA followed by Tukey’s multiple comparisons post hoc test. * *p* < 0.05, ** *p* < 0.01, *** *p* < 0.001, **** *p* < 0.0001 defined as significant.

In view of the pathological role of p53 in AKI [29], we examined the expression and phosphorylation at the site of Ser15 of p53 in kidneys. Control kidneys exhibited very low p53 and p-p53 expression, whereas IRI kidneys displayed markedly increased expression of p53 and p-p53 (Figure 5A,B). Of note, *Lats2* knockout significantly increased the level of p53 and p-p53 (Figure 5A,B). The level of p-MDM2 (Ser186) was higher in the kidneys of *Lats2*-Ctrl mice than in *Lats2*-CKO mice under both sham and IRI conditions, whereas its expression was significantly downregulated after IRI (Figure 5B,D). Immunochemistry staining also revealed p-MDM2 (Ser186) was significantly decreased with *Lats2* deficiency (Figure S3).

We then analyzed the expression of p21, Bax, and cleaved caspase 3, three pro-apoptotic proteins, subjected to p53 regulation in ischemic AKI [30,31,32,33]. As shown in Figure 5B–D, p21, Bax and cleaved caspase 3 were induced by IRI along with a change in p53 in Lats2-Ctrl kidney tissues, which were largely augmented in *Lats2*-CKO tissues. Although Bcl-2 and Bcl-xL, two anti-apoptotic proteins, were significantly upregulated by IRI in *Lats2*-Ctrl kidneys, however, this induction was suppressed in CKO kidneys. Thus, these data suggest that knockout of *Lats2* on proximal tubules induces the expression and activation of p53 and also the downstream of p53 (p21, Bax, and cleaved caspase 3), but decreased the expression of Bcl-2 and Bcl-xL.

### 2.6. Pharmacologic Inhibition of p53 Attenuates Lats2 Knockout-Induced Exacerbation of Kidney Parenchymal Injury and Renal Fibrosis

**Figure 6:**
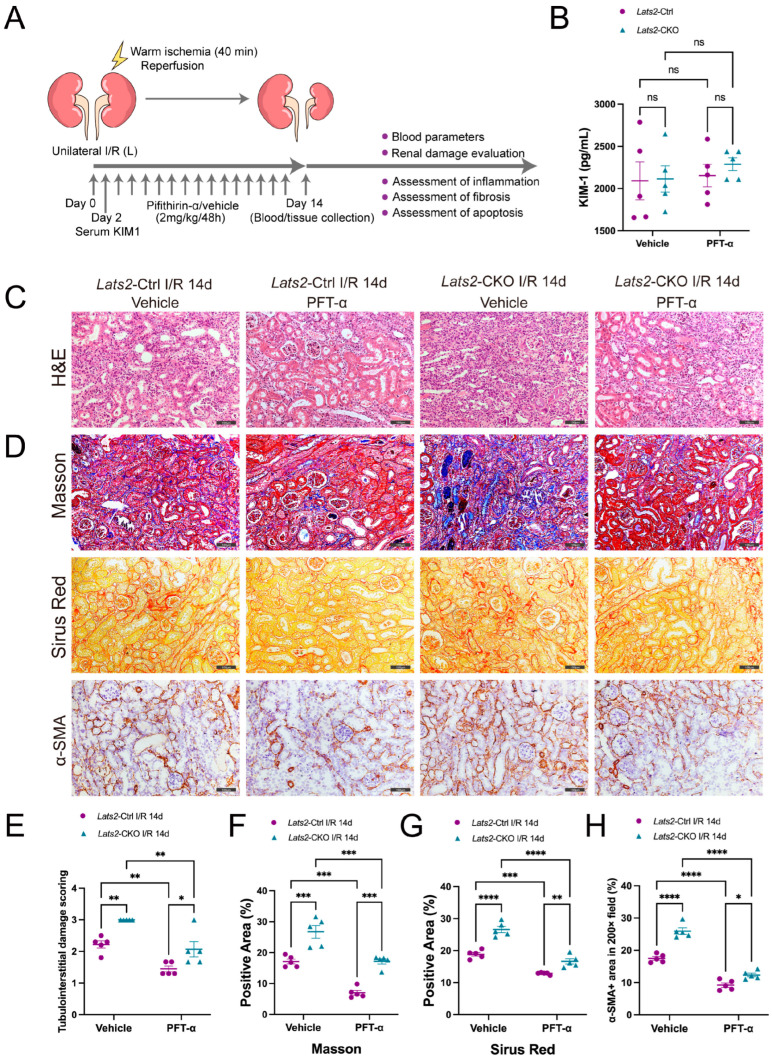
The effect of pifithrin-α on kidney histopathology after I/R injury. (**A**) *Lats2*-Ctrl and *Lats2*-CKO mice were subjected to 40 min warm U-IRI, and then injected with pifithrin-α or control vehicle every 48 h after IRI for 14 days. All mice were euthanized on day 14 after IRI injury. (**B**) Serum KIM-1 levels of mice were determined by ELISA in U-IRI mice with vehicle or PFT-α treatment 2 days after U-IRI (*n* = 5 mice per group). (**C**) Representative HE staining of kidney in U-IRI model of *Lats2*-Ctrl and *Lats2*-CKO mice with vehicle or PFT-α treatment. Scale bars, 100 μm. (**D**) Representative images of Masson, Sirius red, and α-SMA staining of kidney in U-IRI model of *Lats2*-Ctrl and Lats2-CKO mice with vehicle or PFT-α treatment. Scale bars, 100 μm. (**E**) Semi-quantification of tubulointerstitial damage (*n* = 5 mice per group). (**F**) Semi-quantification in renal Sirius red-positive area proportion (*n* = 5 mice per group). (**G**) Semi-quantification in renal Masson-positive area proportion (*n* = 5 mice per group). (**H**) Quantification of α-SMA+ staining (*n* = 5 mice per group). Data expressed as means ± SEM. Two-way ANOVA followed by Tukey’s multiple comparisons post hoc test. * *p* < 0.05, ** *p* < 0.01, *** *p* < 0.001, **** *p* < 0.0001 defined as significant. ns, not statistically significant.

To investigate whether p53 mediated *Lats2*’s function in renal maladaptive repair, we used pifithrin-α (PFT-α, an inhibitor of p53) or vehicle to treat both *Lats2*-Ctrl and *Lats2*-CKO mice after U-IRI and evaluate its long-term outcomes (Figure 6A). The initial proximal tubule injury at 48 h after IRI showed no significant difference after PFT-α treatment between the groups, as determined by serum KIM-1 level (Figure 6B). H&E staining showed that p53 inhibition was able to alleviate kidney parenchymal injury in both *Lats2*-CKO mice and *Lats2*-Ctrl mice caused by U-IRI. However, compared to *Lats2*-Ctrl mice with PFT-α treatment, *Lats2*-CKO mice with PFT-α treatment exhibited more severe renal parenchymal damage (Figure 6C,E). Masson and Sirius red staining demonstrated that PFT-α administration significantly mitigated interstitial fibrosis following AKI in both *Lats2*-CKO and *Lats2*-Ctrl mice, but compared with the *Lats2*-Ctrl group, *Lats2*-CKO kidneys showed more fibrosis after PFT-α injection (Figure 6D,F,G). Similarly, immunochemistry analysis revealed the expression of renal α-SMA, fibronectin and collagen I was notably reduced in PFT-α-treated mice compared to the vehicle group. However, the expression of these fibrosing markers in *Lats2*-CKO IRI kidneys remained higher than those observed in *Lats2*-Ctrl IRI kidneys (Figure 6D,H and Figure S4). These results suggest p53 activation is critical to renal tubulointerstitial damage and fibrosis in response to tubular *Lats2* deficiency after IRI.

### 2.7. In Vivo Inhibition of p53 Alleviates the Aggravation of Renal Inflammatory Cell Infiltration and Cell Apoptosis in Response to Lats2 Deficiency after AKI

**Figure 7:**
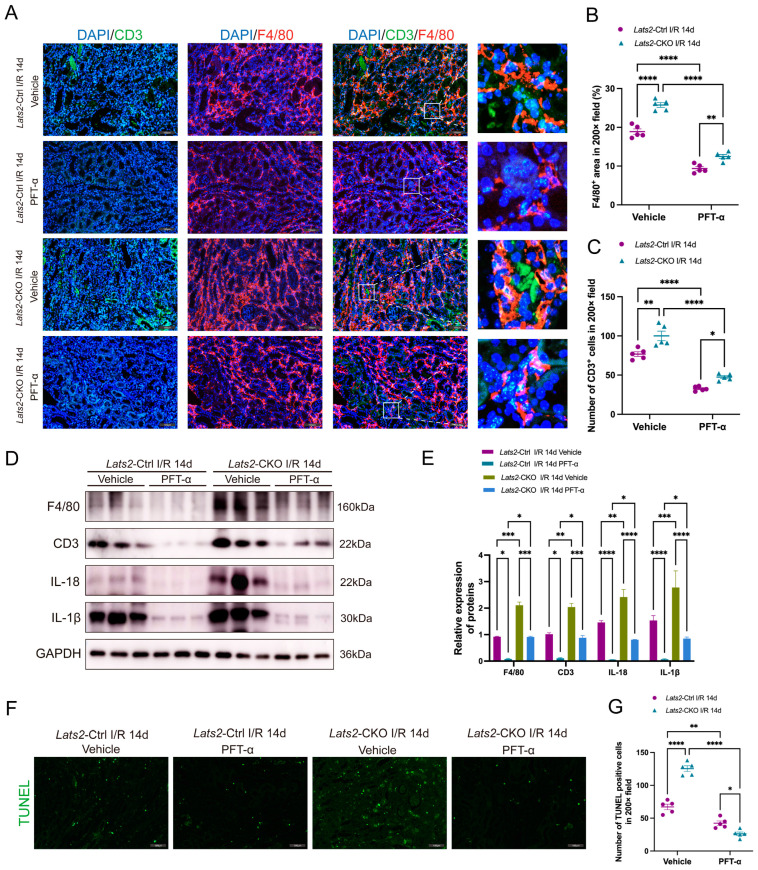
Inhibition of p53 reduces intrarenal inflammatory cell infiltration and cell apoptosis after U-IRI. (**A**) Representative dual fluorescence and enlarged images of CD3 and F4/80 staining in IRI-injured kidneys of *Lats2*-Ctrl and *Lats2*-CKO mice. Scale bars, 100 μm. (**B**) Quantification in renal infiltration of CD3+ T cells (*n* = 5 mice per group). (**C**) Quantification in renal infiltration of F4/80+ macrophages (*n* = 5 mice per group). (**D**) Western blotting analysis of F4/80, CD3, IL-18 and IL-1β in U-IRI model of *Lats2*-Ctrl and *Lats2*-CKO mice with vehicle or PFT-α treatment and (**E**) quantified and normalized to GAPDH expression. (**F**) Representative TUNEL staining of kidney in U-IRI model of *Lats2*-Ctrl and *Lats2*-CKO mice with vehicle or PFT-α treatment. Scale bars, 100 μm. (**G**) Quantification of TUNEL-positive cells in kidney (*n* = 5 mice per group). Data expressed as means ± SEM. Two-way ANOVA followed by Tukey’s multiple comparisons post hoc test. * *p* < 0.05, ** *p* < 0.01, *** *p* < 0.001, **** *p* < 0.0001 defined as significant.

We then examined whether p53 contributed to *Lats2*-CKO mediated inflammatory cell infiltration and cell apoptosis. As shown in Figure 7A–C, PFT-α treatment significantly reduced F4/80- and CD3-positive cell infiltration in the renal interstitium following AKI in both *Lats2*-CKO and *Lats2*-Ctrl mice. Nevertheless, *Lats2*-CKO mice still kept an increased level of inflammatory cell infiltration in kidneys compared to the *Lats2*-Ctrl group (Figure 7A–C). Western blot analysis revealed the expression of F4/80, CD3, IL-18 and IL-1β was significantly decreased after PFT-α treatment in both *Lats2*-Ctrl and *Lats2*-CKO mice with IRI. *Lats2*-CKO mice exhibited higher levels of these proteins than *Lats2*-Ctrl mice, even after the PFT-α treatment (Figure 7D,E). We further examined apoptosis in kidney tissues by TUNEL assay. Notably, much less apoptosis was observed in *Lats2*-CKO and *Lats2*-Ctrl mice after PTF-α intervention, and the number of apoptotic cells in the *Lats2*-Ctrl group was higher than the *Lats2*-CKO group after PFT-α treatment (Figure 7F,G). These results indicate that p53 may be a critical downstream factor participating in kidney inflammation and apoptosis aggravated by *Lats2* ablation.

### 2.8. Lats2 Overexpression Suppressed p53 Expression in TEC Cells Subjected to Sham and Hypoxia/Reoxygenation Treatment

**Figure 8:**
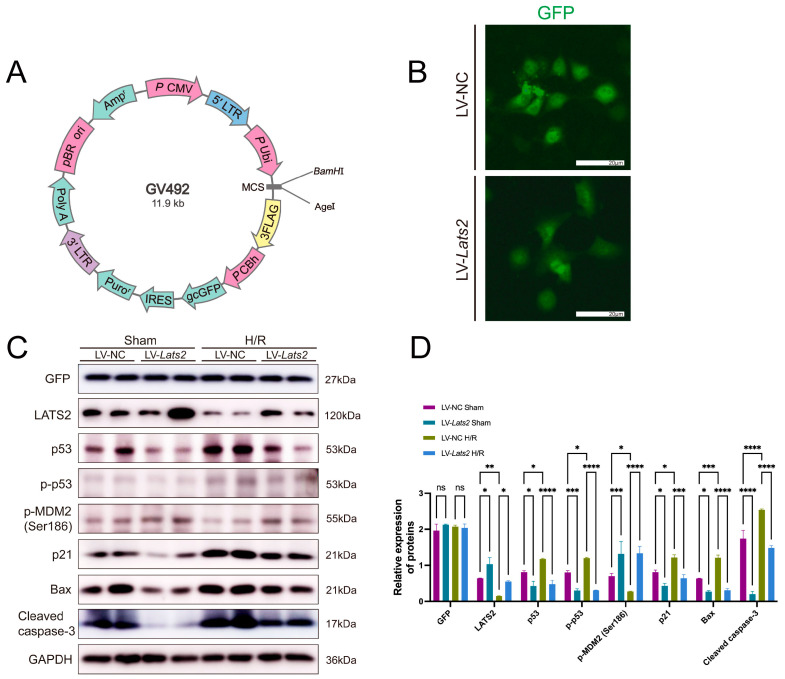
The influence of *Lats2* overexpression on p53 in TEC cells in response to hypoxia and reoxygenation (H/R) injury. (**A**) Vector map of the lentivirus incorporating LATS2 recombinant plasmid. (**B**) Representative images of GFP expression in TEC cells with lentivirus incorporating *Lats2* recombinant plasmid or empty plasmid. Scale bars, 20 μm. (**C**) Western blotting analysis of GFP, LATS2, p53, p-p53, p-MDM2 (Ser186), p21, Bax and cleaved caspase-3 in TEC cells subjected to H/R injury and (**D**) quantified and normalized to GAPDH expression. Data expressed as means ± SEM. Two-way ANOVA followed by Tukey’s multiple comparisons post hoc test. * *p* < 0.05, ** *p* < 0.01, *** *p* < 0.001, **** *p* < 0.0001 defined as significant. ns, not statistically significant.

To further determine the role of LATS2 in p53 expression after AKI, TEC cells were induced to overexpress *Lats2* by lentivirus transfection, hereafter referred to as LV-*Lats2*. Lentivirus incorporating an empty plasmid was transfected into cells to serve as a negative control, hereafter referred to as LV-NC (Figure 8A,B). Equal transduction efficiencies were validated by the uniform expression of GFP in the transfected cells (Figure 8C,D). The transfected cells were then exposed to hypoxia/reoxygenation (H/R) stimulation to mimic IRI in vitro. Western blot showed that expression of p53, p-p53, and other pro-apoptotic makers (p21, Bax and cleaved caspase-3) were upregulated after H/R treatment, but significantly decreased by overexpressing *Lats2* in TECs without or with H/R treatment, indicating *Lats2* overexpression attenuates cellular apoptosis (Figure 8C,D). However, *Lats2* overexpression caused a significant increase in p-MDM2 (Ser186), which is similar to the results in vivo (Figure 8C,D). p-MDM2 (Ser186) is a suppressive molecule promoting ubiquitination degradation of p53. Thus, we demonstrated that upregulation of LATS2 could increase the phosphorylation of MDM2 at the Ser186 site and suppress p53 as well as downstream pro-apoptotic markers.

## 3. Discussion

How LATS2, the upstream factor in the Hippo signaling pathway, functions in acute and chronic kidney diseases remains poorly understood. And whether LATS2 possesses distinct actions in cell cycle regulation and repair processes during AKI, independent of the Hippo pathway, remains a problem to be explored [34]. The present study investigated the potential role of LATS2 in AKI-to-CKD transition, and found firstly that renal LATS2 expression was decreased after AKI. Using *Lats2* conditional knockout mice, we demonstrated that proximal tubule-specific *Lats2* ablation exacerbated ischemic AKI-induced tubular maladaptive repair, leading to more severe tubulointerstitial fibrosis, inflammatory cell infiltration, tubular apoptosis, and upregulation of p53 than control mice. Inhibiting p53 activation attenuated *Lats2* deficiency-induced aggravation of renal inflammation, fibrosis, and apoptosis following ischemic AKI. In vitro, overexpression of LATS2 was further confirmed to negatively regulate p53 expression.

In the Hippo pathway, the knockdown of LATS2 can increase nuclear YAP expression [35]. Nuclear YAP distribution and activation was proved to protect against apoptosis [36,37]. However, in our research, *Lats2* ablation was observed to lead to more apoptosis in kidneys after IRI. Therefore, we postulate the existence of other mechanisms that also play a role in regulating cell apoptosis when *Lats2* is deficient.

Previous reports indicate that nuclear LATS2 is implicated in promoting p53 activation in response to mitotic apparatus or oncogenic stress by preventing MDM2-driven p53 degradation [38]. Within this process is embedded a positive feedback loop, where p53 also positively regulates *Lats2* gene transcription, resulting in a continuous increase in nuclear LATS2 protein levels [39]. This constitutes a LATS2-p53 tumor-suppressing axis that regulates apoptosis through the activation of downstream transcriptional p53-dependent genes [40]. Intriguingly, contrary to some cancer research, our study showed that renal proximal tubule *Lats2* ablation increased cellular apoptosis through upregulation of p53 after severe AKI. Similarly, Qi et al. reported that *Lats2* knockdown exacerbated podocyte apoptosis in diabetic nephropathy [35], which is consistent with our findings. In vitro results also showed that *Lats2* overexpression led to reduced levels of p53 and its downstream substrates under normoxic conditions or with H/R treatment. This suggests that hypoxia stress may not be the primary determinant of the differential relationship outcomes between *Lats2* and p53. Therefore, it is highly probable that the function of LATS2 and its role in p53 regulation may vary depending on the specific cell type and pathological context [41].

A previous study demonstrated that LATS2 targets p53-dependent apoptosis by inhibiting the activity of c-Abl, which leads to increased p53 levels and activity [42]. Thus, in view of the roles of the Hippo pathway components being cell type and state-dependent, it is plausible that LATS2 regulates p53 expression and cell apoptosis through a distinct mechanism in kidney diseases. In the present study, we made an interesting observation regarding p-MDM2 (Ser186), which is known to mediate the ubiquitination degradation of p53 [43]. Specifically, we found that p-MDM2 (Ser186) was downregulated in vivo with *Lats2* knockout and upregulated in vitro with *Lats2* overexpression. A decrease in p-MDM2 (Ser186) in *Lats2*-CKO kidneys can cause p53 accumulation, which provides an explanation of the opposite trend of p53 in AKI kidneys compared to tumor tissues. Further investigation is needed to clarify this issue in AKI.

Compelling evidence supports the pivotal role of p53 in the pathogenesis of AKI and post-AKI kidney repair [44,45,46]. In combination with our results, PFT-α treatment alleviates IRI-induced renal maladaptive repair in both *Lats2*-Ctrl and *Lats2*-CKO mice, further verifying that p53 activation mediates aggravated kidney damage induced by *Lats2* ablation. It should be noted, however, that despite the protective effects of PFT-α on both groups, *Lats2*-CKO mice exhibited a higher degree of fibrosis and inflammation compared to *Lats2*-Ctrl mice in the treated cohort. Evidence suggests that severe AKI can lead to constant YAP activation, which results in maladaptive renal repair and CKD through mechanisms such as macrophage infiltration, epithelial–mesenchymal transition, fibroblast-to-myofibroblast transition, and G2/M-phase cell-cycle arrest [26]. Considering that *Lats2* knockout can lead to increased YAP expression, inhibition of p53 does not completely resist the inflammatory or fibrotic aggravation from LATS2 deficiency, which might be the reason that even in the presence of PFT-α treatment, *Lats2*-CKO mice exhibited severe chronic kidney damage compared to *Lats2*-Ctrl mice, suggesting that LATS2 deficiency exacerbates incomplete repair of AKI via p53-independent mechanisms. It is worth noting that p53 also serves as an autophagy regulator in various forms of AKI [47], and whether LATS2 is involved in autophagy by influencing p53 activation during AKI–CKD transition deserves further research.

Collectively, this study demonstrates that *Lats2* ablation leads to a decrease in p-MDM2 (Ser186) and upregulation of p53 in renal maladaptive repair following severe ischemic AKI. Pharmacological inhibition of p53 can rescue the aggravation of renal maladaptive repair caused by *Lats2* knockout. In vivo, we verified that *Lats2* overexpression can upregulate p-MDM2 (Ser186) and suppress p53, which further indicates LATS2 functions in AKI maladaptive repair via the MDM2-p53 axis. Nevertheless, the mechanism by which LATS2 promotes MDM2 phosphorylation is still unclear and needs further investigation. We propose that targeting LATS2 may facilitate a treatment strategy for AKI-to-CKD transition.

## 4. Materials and Methods

### 4.1. Animals

γG*gt1*-Cre (JAX stock #012841) [48] and *Lats2*^flox/flox^ (JAX stock #025428) [49] mouse lines were obtained from the Jackson Laboratory. *Lats2*^flox/flox^ mice were crossed with *γGgt1*-Cre mice to produce proximal tubular *Lats2* conditional knockout (*Lats2*-CKO) and proximal tubule *Lats2* wild-type (*Lats2*-Ctrl) littermate mice, which are depicted in the breeding protocol in Figure 2A. Eight-week-old male C57BL/6 mice weighing 23–25 g were used in this work. The mice were housed in a specific pathogen-free environment at the Animal Center of the Shanghai General Hospital at the optimal temperature with a 12 h light and 12 h dark cycle. All animal experiments were performed in strict accordance with the guidelines of the National Institutes of Health’s *Guide for the Care and Use of Laboratory Animals* and were approved by the Ethics Committee of Shanghai General Hospital, Shanghai Jiaotong University School of Medicine (Shanghai, China).

### 4.2. RNA-seq

RNA samples were provided to GENEWIZ, Azenta Life Sciences. Total RNA of each sample was quantified and qualified by an Agilent 2100 Bioanalyzer (Agilent Technologies, Palo Alto, CA, USA), NanoDrop (Thermo Fisher Scientific Inc., Waltham, MA, USA) and 1% agarose gel. Library construction was carried out using a VAHTS mRNA-seq V3 Library Prep Kit for Illumina (NR611) through polyA selection. Each sample was amplified by PCR for 13 cycles using P5 and P7 primers, with both primers carrying sequences that can anneal with the flow cell to perform bridge PCR and the P7 primer carrying a six-base index allowing for multiplexing. The PCR products were cleaned up using beads, validated using an Qsep100 (Bioptic, Taiwan, China), and quantified with a Qubit3.0 Fluorometer (Invitrogen, Carlsbad, CA, USA). Then, libraries with different indices were multiplexed and loaded on an Illumina HiSeq instrument according to manufacturer’s instructions (Illumina, San Diego, CA, USA). Sequencing was carried out using a 2 × 150 bp paired-end (PE) configuration; image analysis and base calling were conducted by the HiSeq Control Software (HCS) (v2.2.68) + OLB + GAPipeline-1.6 (Illumina) on the HiSeq instrument.

### 4.3. Bioinformatic Analysis

Reference genome sequences and gene model annotation files of relative species were downloaded from the genome website ENSEMBL. Hisat2 (v2.0.1) was used to index reference genome sequences and align clean data to reference genome. For differential expression analysis, we used the DESeq2 Bioconductor package. For all the analyses, we only kept genes with FDR-transformed *p* values below 0.05, log fold change of at least 0.58, and FPKM above 1 in IRI and/or control samples.

### 4.4. In Vivo Unilateral Renal Ischemia/Reperfusion Model

The U-IRI-induced AKI mouse model was established as previously described [50]. Briefly, the mice were randomly assigned and anaesthetized intraperitoneally with pentobarbital sodium (6 mg/100 g) on a 37 °C warming pad. The renal pedicles were then exposed by a midline incision. Warm renal ischemia was induced by clipping the pedicle of the left kidney for 40 min using a nontraumatic microaneurysm clip (Rivard Life Science Co. Ltd., Shenzhen, China), leaving the right kidney intact. The clamp was released and reperfusion was confirmed visually. Sham control mice underwent the same operation without renal pedicle clamping. During surgery, the body temperature of mice was maintained at 37 °C. Prewarmed saline solution (0.5 mL; 37 °C) was intraperitoneally injected before the abdomen was closed. The mice were euthanized on day 14 after renal surgery (*n* = 5/group). Blood and kidney tissue samples were obtained at the indicated times after the surgery for further analysis.

### 4.5. TEC Cell Culture and Treatment

A murine renal tubular epithelial cell line (TEC; Fuheng Cell Center, Shanghai, China) was cultured in Dulbecco’s modified Eagle’s medium–nutrient mixture F-12 (HyClone Laboratories, Logan, UT, USA) with 10% fetal bovine serum (Thermo Fisher Scientific, Waltham, MA, USA) and 1% penicillin–streptomycin stock solution (Thermo Fisher Scientific). To simulate I/R injury in vitro, we used a model of hypoxia/reoxygenation (H/R). TEC cells were exposed to a hypoxia incubator chamber (STEMCELL Technologies, Vancouver, BC, Canada) with an anoxic mixture gas (95% N_2_ and 5% CO_2_) for 12h at 37 °C. Then, cells were returned to complete growth medium and placed in a normoxic chamber (37 °C, 5% CO_2_) for 2 h of reoxygenation.

*Lats2* was overexpressed via transfecting with a lentivirus incorporating the *Lats2* recombinant plasmid (Ubi-MCS-3FLAG-CBh-gcGFP-IRES-puromycin, GENECHEM, Shanghai, China), referred to as LV-*Lats2*. LV-NC, a lentivirus incorporating an empty plasmid, served as a negative control. Viral particles were removed 16 h later after transfection, and cells were replenished with fresh TEC medium. Puromycin selection commenced 24 h later with the addition of 2 μg/mL puromycin. Surviving cells were isolated into single cell clones. The cells after treatment were lysed in RIPA lysis buffer (Beyotime, Shanghai, China) for western blotting analysis.

### 4.6. Pharmacological Inhibitor

Pifithrin-α (PFT-α) was used as previously described [51] to inhibit p53 activity (Cat #S2929, Selleck, Shanghai, China). Mice after the ischemia/reperfusion (I/R) surgery were intraperitoneally administered with PFT-α (2 mg/kg) or vehicle and every 48 h for 14 days.

### 4.7. Serum Measurements

Mouse blood was collected at the indicated time points. Creatinine (Cr) and blood urea nitrogen (BUN) chemical kits were purchased from Jiancheng Bioengineering Institute of Nanjing (Nanjing, China). Serum kidney injury molecule 1 (KIM-1) concentrations were measured using the mouse KIM-1 ELISA Kit (J&L Biological, Shanghai, China). Experiments were performed according to the manufacturer’s protocol.

### 4.8. Western Blotting

Protein was extracted from kidney tissue as previously described [9]. Briefly, 45 μg protein of each sample underwent 10% SDS-PAGE electrophoresis and was then transferred to a PVDF membrane. Nonspecific binging sites of the membrane were blocked with 5% nonfat milk in Tris-buffered saline containing 0.1% Tween. After that, the membrane was incubated with the following primary antibodies at 4 °C overnight: polyclonal rabbit anti-LATS2 (Cat# 20276-1-AP, Proteintech, Hong Kong, China), polyclonal rabbit anti-Phospho-LATS2 (ser872) (Cat# AF7440, Affinity), monoclonal mouse anti-p53 (Cat# sc-126, Santa Cruz, Santa Cruz, CA, USA), monoclonal rabbit anti-p-p53 (Ser15) (Cat# 9284S, CST), monoclonal mouse anti-p21 (Cat# sc-6246, Santa Cruz), monoclonal rabbit anti-Bax (Cat# ab32503, Abcam, Cambridge, UK), polyclonal rabbit anti-cleaved caspase-3 (Cat# AF7022, Affinity), monoclonal rabbit Bcl-xL (Cat# 2764S, CST), monoclonal mouse anti-Bcl-2 (Cat# sc-7382, Santa Cruz), monoclonal rabbit anti-α-SMA (Cat# 19245S, CST), polyclonal rabbit anti-fibronectin (Cat# 15613-1-AP, Proteintech), polyclonal rabbit anti-collagen I (Cat# 14695-1-AP, Proteintech), monoclonal rabbit anti-F4/80 (Cat# 70076S, CST), monoclonal rabbit anti-CD3 (Cat# 99940S, CST), monoclonal rabbit anti-IL-18 (Cat# 57058S, CST), monoclonal rabbit anti-IL-1β (Cat# 12426, CST), polyclonal rabbit anti-phospho-MDM2 (Ser186) (Cat# bs-5471R, Bioss, Woburn, MA, USA), monoclonal mouse anti-β-actin (Cat# sc-47778, Santa Cruz), and monoclonal mouse anti-GAPDH (Cat# 60004-1-1g, Proteintech). ImageJ (V1.52, National Institutes of Health, Bethesda, MD, USA) was used to quantify blot images. Expression level of target proteins was normalized to that of β-actin expression level.

### 4.9. Kidney Histopathology and TUNEL Assay

Formalin-fixed, paraffin-embedded sections (2 µm) of kidneys were subjected to hematoxylin and eosin (H&E), Sirius red (SR), and Masson trichrome staining. H&E staining was assessed in a blinded fashion in 10 consecutive fields at a magnification of 200× per section. The severity of tubulointerstitial damage was graded from 0 to 3 according to the distribution of lesions: 0, no lesion; 1, less than 20%; 2, 20–50%; 3, more than 50% [52]. Positive areas of Masson trichrome (blue) and Sirius red (red) staining were evaluated at 200× magnification using ImageJ (National Institutes of Health, Bethesda, MD, USA) to determine ECM deposition. TUNEL assay was conducted using TUNEL BrightGreen Apoptosis Detection Kit (Vazyme Biotech, Nanjing, China). For quantification, 10 fields were randomly selected from each tissue section under 200× magnification.

### 4.10. Immunofluorescence and Immunohistochemistry

We performed immunostaining as previously described [9,52]. Kidneys were fixed in 4% paraformaldehyde, embedded in paraffin, and cut into 2 μm-thick sections. F4/80-, CD3-, p53- and p-p53-positive cells were detected by immunofluorescence using monoclonal rabbit anti-F4/80 (Cat# 70076S, CST), monoclonal rabbit anti-CD3 (Cat# 99940S, CST), monoclonal mouse anti-p53 (Cat# sc-126, Santa Cruz) and monoclonal rabbit anti-p-p53 (Ser15) (Cat# 9284S, CST). Fluorescent images were taken using a Zeiss Axioplan-2 imaging microscope. For immunochemistry, α-SMA, fibronectin, and collagen I were detected using monoclonal rabbit anti-α-smooth muscle actin (Cat# 19245S, CST), polyclonal rabbit anti-fibronectin (Cat# 15613-1-AP, Proteintech), polyclonal rabbit anti-collagen I (Cat# 14695-1-AP, Proteintech), and polyclonal rabbit anti-phospho-MDM2 (Ser186) (Cat# bs-5471R, Bioss). Ten non-overlapping, randomly chosen fields were analyzed per kidney under 200× magnification, and the percentage area of positive staining was quantified using ImageJ (National Institutes of Health, Bethesda, MD, USA).

### 4.11. Statistics

Statistical analyses were performed using GraphPad 9.4.1 software. Study groups were analyzed by two-way ANOVA using Tukey’s multiple comparisons post hoc test. Data are presented as means ± SEM. *p* values < 0.05 were considered statistically significant.

## Data Availability

Representative data are publicly archived datasets. For further information, please contact the corresponding author.

## Supplementary Materials

The following supporting information can be downloaded at https://www.mdpi.com/article/10.3390/ijms242015258/s1.
