# Supplementary material for: Proximal Tubular Lats2 Ablation Exacerbates Ischemia/Reperfusion Injury (IRI)-Induced Renal Maladaptive Repair through the Upregulation of P53
Source: Int J Mol Sci. 2023 Oct 17;24(20):15258. doi: 10.3390/ijms242015258 (PMC10607662; doi:10.3390/ijms242015258)
Supplement: Supplementary file 1 [file ijms-24-15258-s001.zip › ijms-2624614-supplementary.pdf]

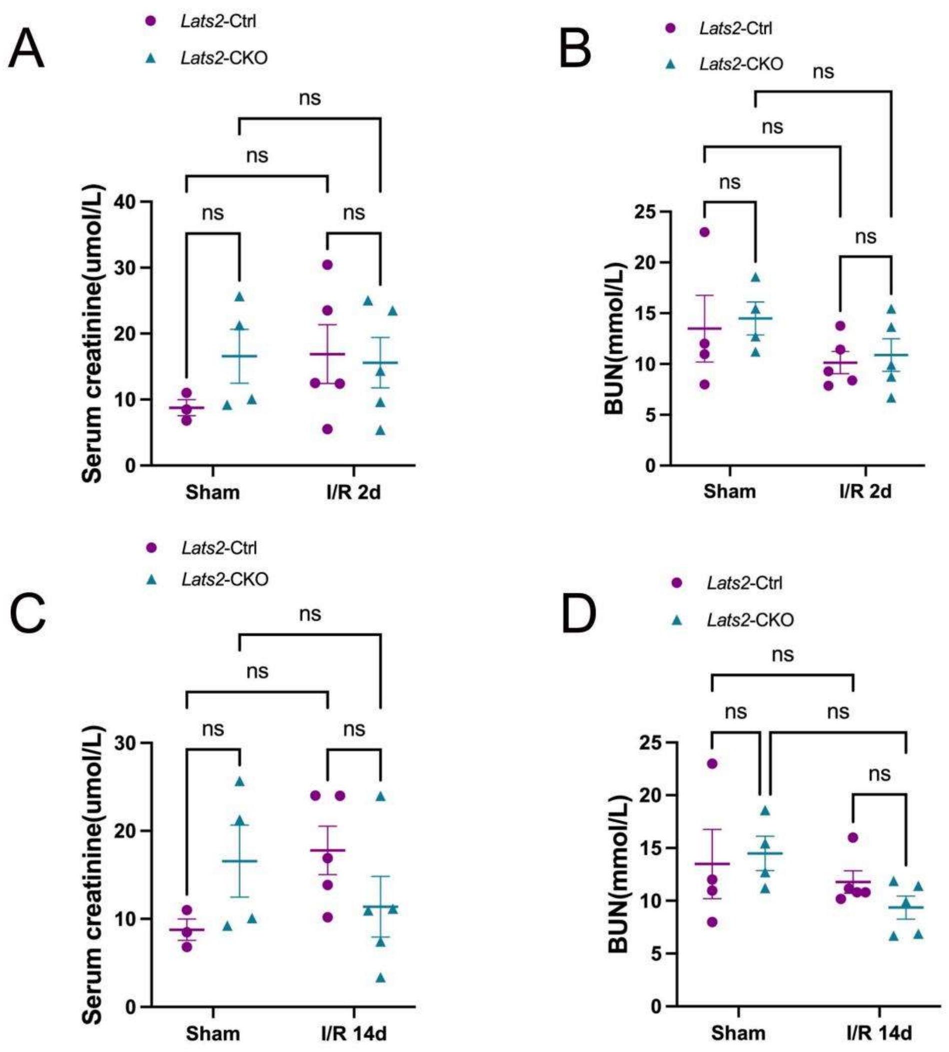

Figure S1. Impact of U-IRI on renal function. (A) Serum creatinine and (B) blood urea nitrogen levels of *Lats2*-Ctrl and *Lats2*-CKO mice at day 2 after IRI (sham n=4, I/R 14d n=5). (C) Serum creatinine and (D) blood urea nitrogen levels of *Lats2*-Ctrl and *Lats2*-CKO mice at day 14 after IRI (sham n=4, I/R 14d n=5). Data expressed as means±SEM. Two-way ANOVA followed by Tukey's multiple comparisons post hoc test. ns, not statistically significant.

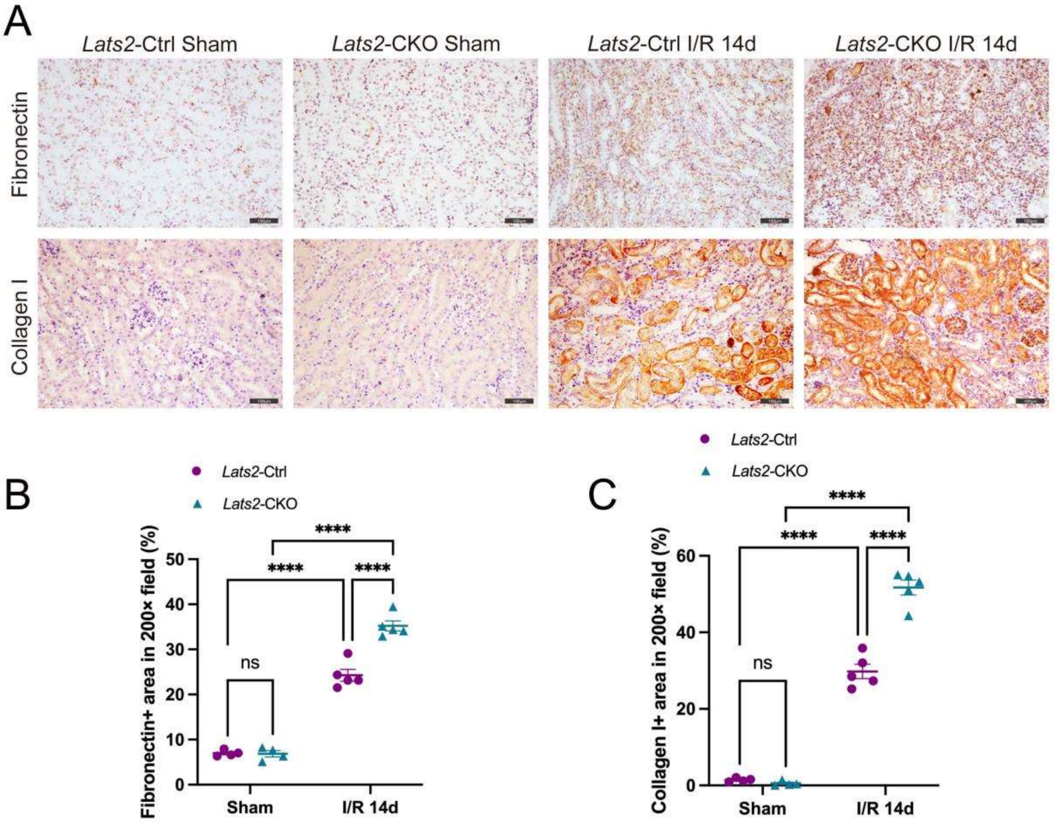

Figure S2. The impact of renal proximal tubule-specific *Lats2* ablation on Fibronectin and Collagen I expression after IRI. (A) Immunochemistry staining of Fibronectin and Collagen I in sham and I/R 14d mice. Scale bars, 100 μm. (B) Semi-quantification of Fibronectin+ staining (sham n=4, I/R 14d n=5). (C) Semi-quantification of Collagen I+ staining (sham n=4, I/R 14d n=5). Data expressed as means±SEM. Two-way ANOVA followed by Tukey's multiple comparisons post hoc test. *****P*<0.0001 defined as significant. ns, not statistically significant.

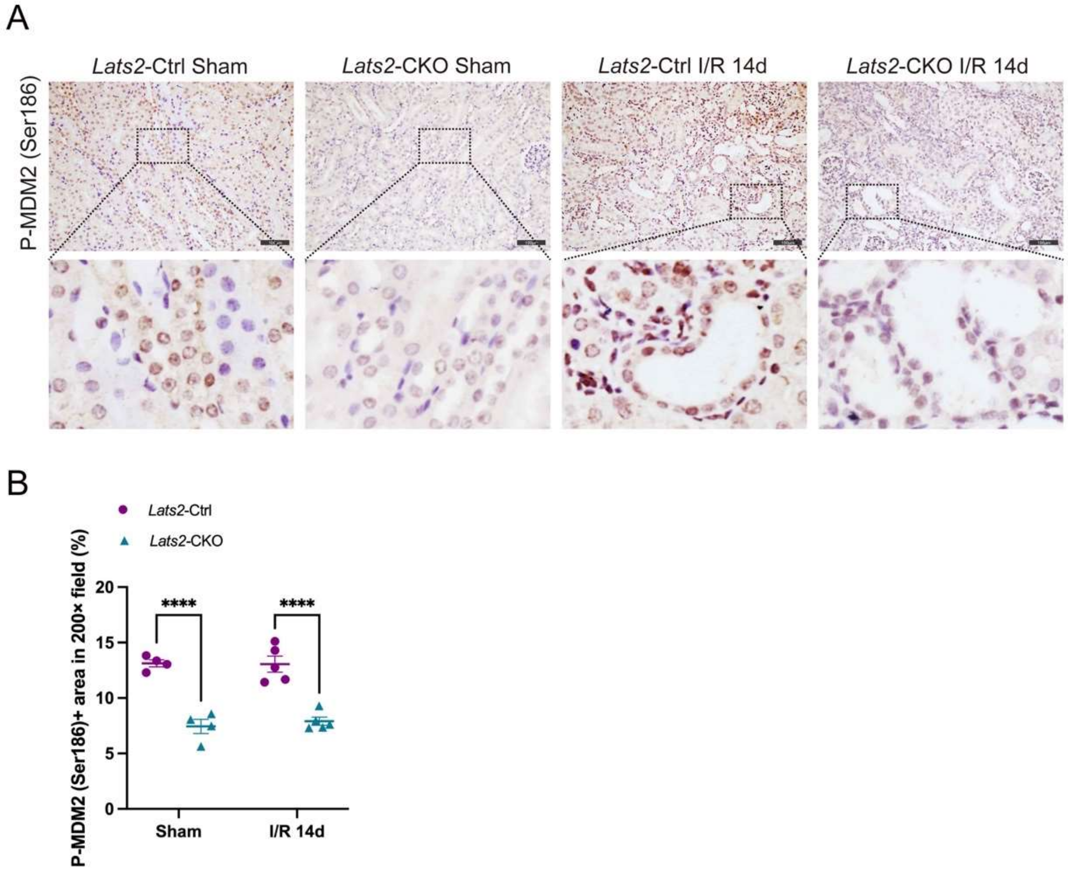

Figure S3. The impact of renal proximal tubule-specific *Lats2* ablation on P-MDM2 (Ser186) expression after IRI. (A) Immunochemistry staining of P-MDM2 (Ser186) staining in sham and I/R 14d mice. Scale bars, 100 μm. (B) Semi-quantification of P-MDM2 (Ser186) staining (sham n=4, I/R 14d n=5). Data expressed as means±SEM. Two-way ANOVA followed by Tukey's multiple comparisons post hoc test. ****P<0.0001 defined as significant.

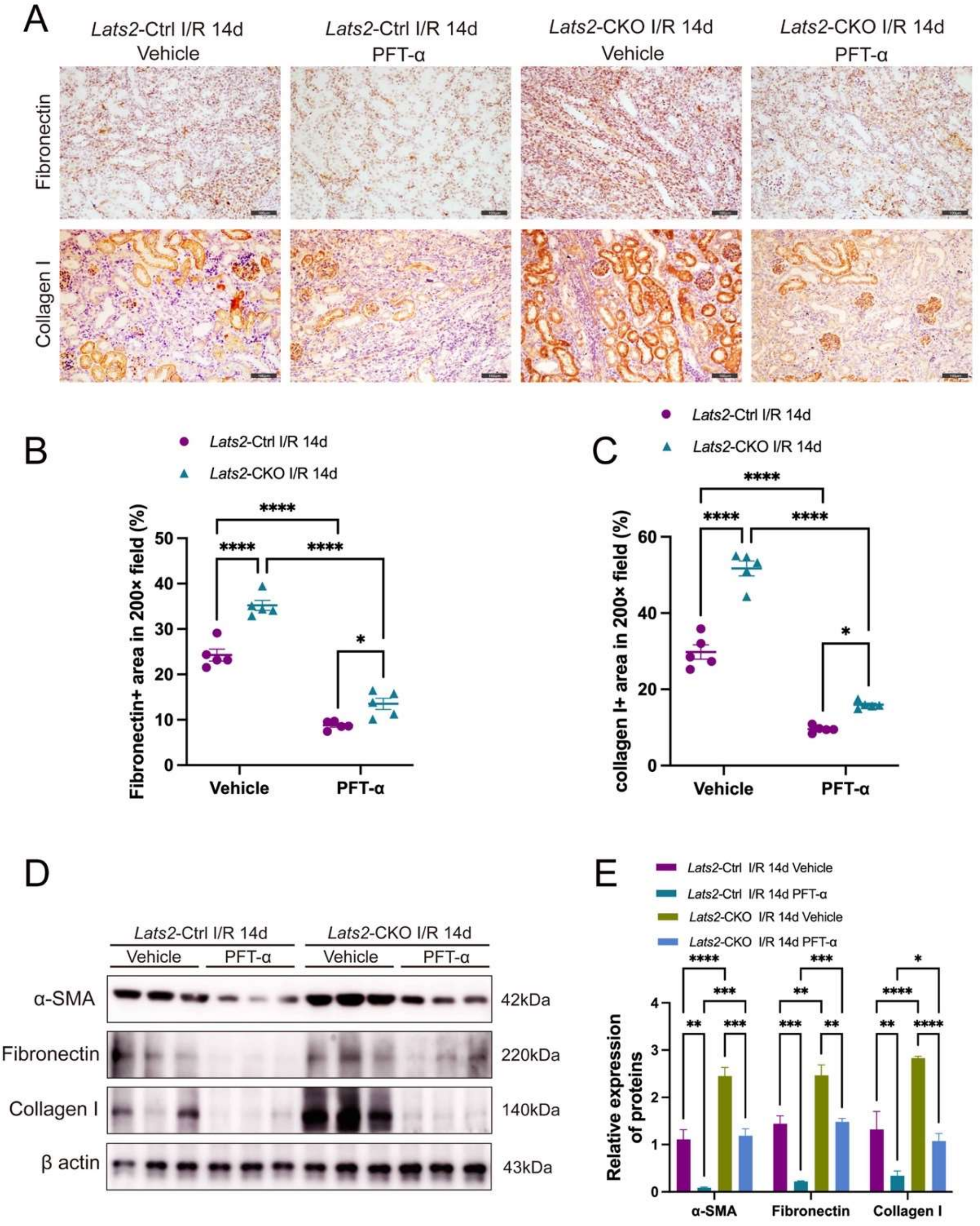

Figure S4. The impact of pifithrin-α on renal α-SMA, Fibronectin and Collagen I expression after IRI. (A) Immunochemistry staining of Fibronectin and Collagen I in U-IRI model of *Lats2*-Ctrl and *Lats2*-CKO mice with vehicle or PFT-α treatment. Scale bars, 100 μm. (B) Semi-quantification of Fibronectin+ staining (n=5 per group). (C) Semi-quantification of Collagen I+ staining (n=5 per group). (D) Western blotting analysis of α-SMA in U-IRI model of *Lats2*-Ctrl and *Lats2*-CKO mice with vehicle or PFT-α treatment and (E) quantified and normalized to β-actin expression. Data expressed as means±SEM. Two-way

ANOVA followed by Tukey’s multiple comparisons post hoc test. *$P<0.05$, **$P<0.01$, ***$P<0.001$, ****$P<0.0001$ defined as significant.
